# Biochar Remediation Improves the Leaf Mineral Composition of *Telfairia occidentalis* Grown on Gas Flared Soil

**DOI:** 10.3390/plants7030057

**Published:** 2018-07-13

**Authors:** Doris Akachukwu, Michael Adedapo Gbadegesin, Philippa Chinyere Ojimelukwe, Christopher John Atkinson

**Affiliations:** 1 Department of Biochemistry, Michael Okpara University of Agriculture, Umudike, Umuahia P.M.B 7267, Abia State, Nigeria; 2 Department of Biochemistry, Faculty of Basic Medical Sciences, University of Ibadan, Ibadan 90001, Oyo State, Nigeria; magbadegesin@yahoo.com; 3 Department of Food Science and Technology, Michael Okpara University of Agriculture, Umudike, Umuahia P.M.B 7267, Abia State, Nigeria; philippaco60@gmail.com; 4 Department of Agriculture, Health and Environment, Natural Resources Institute, University of Greenwich, Chatham ME4 4TB, UK; C.J.Atkinson@gre.ac.uk

**Keywords:** biochar, gas flare, mineral content, nutritional content, protein, soil remediation, vitamin

## Abstract

This study evaluates the effects of remediation of gas flared soil by biochar on the nutritional composition of cultivated *Telfairia occidentalis* leaves, relative to non-gas flared soil. Gas flared soils are degraded due to the presence of heavy metals, noxious gases, carbon soot and acidic rain. Biochar produced from oil palm fibre was applied at five different amounts: 0 t ha^−1^, 7.1 t ha^−1^, 13.9 t ha^−1^, 20.9 t ha^−1^ and 28.0 t ha^−1^ to containerized soils (both gas flared and control soil), inside a greenhouse, which were allowed to mineralize for two weeks. Two viable seeds of *T. occidentalis* per replicate were sown. After eight weeks of growth, leaves were harvested, dried and chemically analyzed. Application of biochar significantly increased leaf ash and crude fibre content of *Telfairia occidentalis*. Plants from soil treated with 13.9 t ha^−1^ of biochar had the highest concentrations of vitamins A, B_1_, B_2_, B_6_, C and E irrespective of soil type. Maximum increase in leaf vitamin and mineral content was obtained from leaves cultivated on gas flared soil treated with 13.9 t ha^−1^ and 7.1 t ha^−1^ of biochar respectively. The results show that biochar treatment can increase leaf mineral concentrations and that this effect is dependent on the amount of biochar application.

## 1. Introduction

Crude oil accounts for more than 50% of the global energy supply [1]. The process of energy generation derived from crude oil produces numerous undesirable environmental impacts such as oil spills, venting and flaring [2]. Gas flaring is the burning off of associated natural gas during oil exploration [3]. Gas flaring is a usual occurrence in countries that lack the infrastructure to capture, store, utilize, or market natural gas like Nigeria. Nigeria flares more than 70% of associated natural gas from over 120 flaring sites in the Niger Delta. This has earned Nigeria the status of one of the highest emitters of greenhouse gases (GHG) worldwide [4]. Several campaigns by the World Bank have failed to have an impact on this statistic, and gas flaring continues to increase. In 2011, Global Gas Flaring Reduction [5] reported a 2 billion m^3^ increase in gas flaring since 2010.

Anomohanran [6] reported that the thermal effects of gas flaring could be detected over 2 km from the flare site. Flaring releases pollutants such as greenhouse gases, volatile organic compounds, polyaromatic hydrocarbons, black carbon and particulate matter [7], and heavy metals [8]. These all have serious negative impacts on climate, vegetation, animals and humans [9]. Greenhouse gases such as CO_2_ and methane, are key contributors to climate change [10]. Some atmospheric pollutants from gas flares, such as oxides of nitrogen, carbon and sulphur (NO_2_ CO_2_, CO and SO_2_), particulate matter and hydrogen sulphide (H_2_S) acidifies and depletes the soil of its nutrients [11]. Acidic pH negatively affects the growth and survival of many terrestrial plants and crops as well as aquatic life [12]. Orimoogunje et al. [13] reported that the soils around gas flare sites were rapidly losing their fertility. This loss of soil fertility was due to acid rain, arising from the interaction between gas combustion products, and atmospheric moisture which has been suggested to be responsible for the abandonment of farmlands [14].

The intense heat released during flaring can prevent the growth of vegetation around the flare areas, causing scotching and stunted plant growth. Increased temperatures also impact on the presence and activity of microorganisms within the soil which determine soil health and function, via processes such as the breakdown of organic matter and nitrogen fixation [15].

Reports by Imevbore and Adeyemi [16] show that gas flaring reduced the nutritional quality of crops. Low yam and cassava yields have also been reported within a 2 km radius of a flare site [17]. Consumption of leafy green vegetables, particularly *Telfairia occidentalis*, is very common in Nigeria. *Telfairia occidentalis* is a rich source of minerals, vitamins, protein and phytochemicals [18]. The nutritional value of plants can be negatively affected by the presence of toxic compounds in the soil and atmosphere [19].

Techniques such as the use of chemicals, microorganisms, and plants have been exploited to remediate polluted soils [20]. Some of these approaches have the potential to further contaminate the environment, hence the need for the search for natural and more environmentally sustainable soil remediation approaches. Biochar is the product of pyrolysis of biomass in limited or no oxygen supply [21,22]. It is derived from recalcitrant carbonized organic matter that is often produced from agricultural wastes [23], such as plant biomass and excreted animal waste. It has the potential to increase crop yield, reduce GHG emissions, thereby mitigating climate change, as well as increasing long-term soil carbon sequestration [22]. Biochar application can improve soil aeration [24] and water retention [25], and reduce nutrient leaching [26]. However, the effect of biochar depends on soil type, local environmental conditions, type of biochar used and the temperature of pyrolysis of biochar used. The study here has been conducted to compare and evaluate the effects of biochar remediation, using different rates of application, on the nutritional quality of *T. occidentalis* (commonly known as fluted pumpkin) cultivated on a gas flared soil and non-gas flared soil. A range of vitamins and minerals have been measured to provide insight into the possible changes, induced by gas flaring on the availability of vitamins and minerals provided by the consumption of *T. occidentalis*. 

## 2. Results and Discussion

### 2.1. Chemical Composition of Biochar and Soils

The chemical composition of the biochar produced from oil palm bunch is shown in (Table 1). Palm bunch biochar contained 1.35% nitrogen, 3.02% calcium, 0.80% magnesium, 1.01% potassium, 0.12% phosphorus and 4.05 mg kg^−1^ of sodium. These values were generally lower than that reported for wheat and rice straw biochar [27]. Table 2 depicts the chemical composition of the soils used. Concentrations of phosphorus, nitrogen, organic carbon, organic matter, calcium, potassium, exchangeable acidity and effective cation exchange capacity of the control soil were higher than that of the gas flared soil. 

### 2.2. Effect of Biochar Remediation on the Vitamin Composition of T. occidentalis

The effect of biochar remediation on the vitamin composition of *T. occidentalis* cultivated on non-gas flared soil is shown in Table 3. The 20.9 t ha^−1^ biochar treated plants had the highest composition for all the vitamins. Vitamin compositions of the leaves grown with 13.9 t ha^−1^ and 28.0 t ha^−1^ of biochar were similar. The 7.1 t ha^−1^ biochar treated plants had the lowest vitamin content. The effect of biochar remediation on leaf vitamin concentrations of *T. occidentalis* cultivated on gas-flared soil showed that plants grown with 13.9 t ha^−1^ of biochar had the highest composition of vitamins A, B_1_, B_2_, B_6_, C and E while the control plants had the least (Table 3). Plants grown with the 7.1 t ha^−1^, 20.9 t ha^−1^ and 28.0 t ha^−1^ of biochar had comparable vitamin compositions. The result indicates that the 13.9 t ha^−1^ of biochar produced the maximum vitamin content in *T. occidentalis* plants. Vitamin (A, B_1_, B_2_, B_6_, C and E) leaf concentrations for the non-biochar treated soils from non-gas flared and gas flared sites were all higher than that reported by Uraku et al. [28] for *T. occidentalis* cultivated in a non-gas flaring site in Ebonyi State Nigeria. This observation suggests that the soils (non-gas flared and gas flared) used in our study were more fertile and their fertility can explain the increased vitamin concentrations of *T. occidentalis*. High vitamin concentrations for the 13.9 t ha^−1^, 20.9 t ha^−1^ and 28.0 t ha^−1^ biochar treated *T. occidentalis* leaves compared to 0 t ha^−1^ and 7.1 t ha^−1^ treated group, for both the non-gas flared and gas flared soils suggests that biochar can positively influence vitamin synthesis in *T. occidentalis* leaves.

The differences in vitamin composition between *T. occidentalis* plants cultivated on gas flared (Egbema) and non-gas flared (Umudike) soils showed no statistical differences in any of the vitamin concentrations measured in *T. occidentalis* from the untreated gas flared and non-gas flared soils (Table 3). The vitamin concentrations of *T. occidentalis* grown on gas flared remediated soil were higher than those on non-gas flared soil for 7.1 t ha^−1^ and 13.9 t ha^−1^ biochar treated plants, suggesting that 13.9 t ha^−1^ biochar treatment promoted vitamin synthesis. The result agrees with that of Lou et al. [29] who reported increased vitamin C concentration in cabbage grown with an aqueous extract of maize straw biochar.

### 2.3. Effect of Biochar Remediation on the Proximate Composition of T. occidentalis

The effect of biochar remediation on the proximate composition of *T. occidentalis* cultivated on non-gas flared soil is shown in Table 4. The percentage protein concentration was highest for 7.1 t ha^−1^ (19.1%) biochar treated plants but not significantly (*p* > 0.05) different from 28.0 t ha^−1^ biochar treatment. The 13.9 t ha^−1^ biochar treated and control treatments had the lowest protein content of 12.6%. Lou et al. [30,31] reported a dose dependent increase in soluble protein in cabbage treated with up to a 50x dilution rate of aqueous extract of wheat straw biochar. The 7.1 t ha^−1^ and 28.0 t ha^−1^ biochar treatments showed similar leaf moisture contents. Moisture content was highest for 13.9 t ha^−1^ treatment. The ash and crude fibre content for the 13.9 t ha^−1^, 28.0 t ha^−1^ treatments and the control were similar but significantly (*p* < 0.05) different from 7.1 t ha^−1^ and 20.9 t ha^−1^. The fat content of 7.1 t ha^−1^ treatment was the highest (7.5%) while 28.0 t ha^−1^ treatment was the lowest at 2.1%. Plants treated with 13.9 t ha^−1^ had similar fat contents compared to the control. Carbohydrate content of the treatment groups: 7.1 t ha^−1^, 13.9 t ha^−1^, 20.9 t ha^−1^ and 28.0 t ha^−1^ were similar but statistically (*p* < 0.05) lower than the controls. Increased temperatures also have an impact on the presence and activity of microorganisms within the soil that determine soil health and function, via processes such as the breakdown of organic matter and nitrogen fixation; this may be due to insufficient nutrition that could increase carbohydrate formation [30].

The effect of biochar remediation on the proximate composition of *T. occidentalis* cultivated on gas flared soil showedthat 7.1 t ha^−1^ biochar treated plants had the highest protein content of 18.8%, while the control had the least, 11.8% (Table 4). Protein content of the leaves treated with 13.9 t ha^−1^, 20.9 t ha^−1^ and 25.0 t ha^−1^ of biochar were comparable. Protein and moisture content of the 7.1 t ha^−1^ treated plants were significantly (*p* < 0.05) higher than the control. Ash content was similar for the 7.1 t ha^−1^ and 28.0 t ha^−1^ biochar treatments, while the 13.9 t ha^−1^, 20.9 t ha^−1^ and 0 t ha^−1^ (control) treatments were statistically different. The 7.1 t ha^−1^ biochar treatment had the highest (16.5%) but similar crude fibre content with 13.9 t ha^−1^ biochar, while 28.0 t ha^−1^ treatment had the least (9.9%). The fat content was highest in 20.9 t ha^−1^ treated plants (5.9%), but was similar for 13.9 t ha^−1^ and control treatments. Plants treated with 7.1 t ha^−1^ biochar had the lowest fat content (3.4%). Crude fibre content of 13.9 t ha^−1^ and 28.0 t ha^−1^ biochar treated plants were comparable, while 20.9 t ha^−1^ had the highest carbohydrate content of 54%, but was similar to the control.

A comparative study of the effect of biochar remediation on the proximate composition of *T. occidentalis* cultivated on gas flare and non-gas flare soils (Table 4) showed that protein composition of 28.0 t ha^−1^ treated plants from the gas flared site soil was significantly lower than the control. Moisture content of leaves from the gas flared site was significantly greater for 7.1 t ha^−1^, 28.0 t ha^−1^ and control treatments. Ash and crude fibre concentrations were significantly (*p* < 0.05) different for 7.1 t ha^−1^, 13.9 t ha^−1^, 20.9 t ha^−1^ and control treatments for both soils. Fat content was significantly higher for 13.9 t ha^−1^, 20.9 t ha^−1^, 28.0 t ha^−1^ and control treatments in the gas flared plants, compared to the non-gas flared plants. Proximate analysis was used to assess the nutritional value and quality of this material [31]. Dietary proteins are necessary for the synthesis and maintenance of body tissues, enzymes and hormones [32]. Our result suggest that the vegetables cultivated with 7.1 t ha^−1^ biochar had the highest protein content, and if consumed could be a good source of protein. High ash content in foods indicates high mineral content [33]. The leaves from 7.1 t ha^−1^ biochar treated group were low in mineral content. Consumption of these leaves may not favour maximum mineral supply in the body hence there is need for additional sources of mineral. A high dietary fat content is usually undesirable because it can lead to cardiovascular disorders [34]. The lowest levels of fat were for leaves grown in 7.1 t ha^−1^ biochar treatment. Low carbohydrate levels in 7.1 t ha^−1^ biochar treated leaves suggest that this is sufficient to support normal health and could be a nutritional benefit in reducing the high serum lipid values of diabetic patients [35].

### 2.4. Effect of Biochar Remediation on the Mineral Composition of T. occidentalis

Effect of biochar remediation on the mineral composition of *T. occidentalis* cultivated on non-gas flared soil is shown in Table 5. There were no significant differences in Ca, Mg, K, P or Na concentrations in the biochar treatments compared to the control. Nitrogen, Ca, K, and Na concentrations of 7.1 t ha^−1^ treated *T. occidentalis* leaves were significantly higher than the controls (Table 5). Comparative study of the effects of biochar application on the mineral composition of *T. occidentalis* cultivated on gas flared and non-gas flared soils showed that the nitrogen concentrations were all higher than the control with 7.1 t ha^−1^ treatments being significant (Table 5). The result suggests that increased concentrations of biochar treatment possibly reduced nitrogen uptake in the plants which could decrease protein synthesis and hence supply of essential amino acids to the body [36]. Calcium, Mg and Na concentrations of 7.1 t ha^−1^ and 13.9 t ha^−1^ biochar treated plants were significantly higher compared to the controls with maximal concentrations obtained for the 7.1 t ha^−1^ treatment, indicating that consumption of these plants provides a source of Ca, Mg and Na required for human health [37,38,39]. Potassium and phosphorus concentrations of the plants treated with 7.1 t ha^−1^, 13.9 t ha^−1^, 20.9 t ha^−1^ and 28.0 t ha^−1^ biochar were all significantly higher than the control. Potassium-rich diets have been shown to improve blood pressure control, which impacts positively on the health of the kidney [40]. Phosphorus is necessary for the generation of adenosine triphosphate (ATP) and therefore boosts energy [41]. The findings of this study are in agreement with that of Lou et al. [29], who reported increased potassium, calcium and magnesium in cabbage treated with aqueous extract of Sanli wheat straw biochar. The results of this study suggest that soil enrichment with biochar could positively enhance the mineral composition of *T. occidentalis*, depending on the amount of biochar added to the soil.

## 3. Materials and Methods

### 3.1. Plant Material, Soil Sampling, Plant Growth and Harvesting

*Telfairia occidentalis,* commonly known as fluted pumpkin, or fluted gourd is a member of the family of *Cucurbitaceae*. It was chosen for this study because of its short maturation period of about 3 weeks. Seeds of *T. occidentalis* were obtained locally in Ndoro, Ikwuano L.G.A Abia State, Nigeria.

Soil samples were collected with the aid of a sterile soil auger from a depth of between 0–20 cm. Samples were stored in jute bags and transported to the greenhouse of the National Root Crops Research Institute, Umudike. The soil samples were collected in April from farm land (longitude N 05°33.5′ and latitude E 06°45.2′) close to a flare site (longitude N 05°32.7′ and latitude E 06°45.8′) in Obiakpu Autonomous Community, Ohaji/Egbema L.G.A, Imo State. The control soil, of a comparative type, was collected from farmland where there was no gas flaring activity located at (Longitude N 05°28.513′ and Latitude E 007°32.536′) Umudike, Ikwuano L.G.A, Abia State, Nigeria. The two soils are of the same textural class (sandy loam). The sand fraction was 77.8%, silt 7.8% and clay 14.3% for the gas flared soil while that of the non-gas flared soil was 74.1%, 6.3% and 19.5%, respectively. Soil samples were weighed into plastic containers and mixed with different amounts of biochar, namely 0 t ha^−1^, 7.1 t ha^−1^, 13.9 t ha^−1^, 20.9 t ha^−1^ and 28.0 t ha^−1^ respectively. Water was added to the mixture and the soil allowed to mineralize for two weeks (at a temperature of 29 °C and relative humidity of 75%). Subsequently, two viable seeds of *T. occidentalis* were planted per container in August, 2016 and the experiment was repeated three times with three replicates per experiment. Plants were watered once every two days. Eight weeks after germination, leaves harvested from the same treatment in the 3 replicates were bulked together, thereafter air-dried for 5 days and dried in the oven to a constant weight at 60 °C. 

### 3.2. Biochar Production

Palm bunches obtained as waste products after oil palm processing were collected from oil processing mill and sun-dried until brittle. Biochar was produced at a temperature of 450 °C in a pyrolysis drumkiln. Subsequently, once cool, it was milled to smaller/finer particles and sieved through a 3 mm^2^ mesh and kept dry in a jute bag until used [42].

### 3.3. Determination of Chemical Composition of Palm Bunch Biochar and Soils

Chemical composition of biochar used was determined using inductively coupled plasma optical emission spectrometer (ICP-OES) (Optima 8000, Perkin Elmer, Waltham, MA, USA). Soil pH, exchangeable bases (Ca, Mg, K and Na), exchangeable acidity, nitrogen, organic carbon were determined according to the method of Thomas [43], Udo et al. [44], McLean [45], Bremner and Mulvaney [46] and Nelson and Sommers [47]. Organic matter was derived by multiplying organic carbon value by a factor of 1.724.

### 3.4. Determination of Leaf Proximate and Vitamin Concentrations

The leaf protein, moisture content, ash, crude fibre, fat and carbohydrate composition was estimated according to the method of AOAC [48]. Vitamin concentrations were estimated by the use of HPLC (Waters 616/626 LC System, USA). A quantity of 1.5 g of plant sample was mixed with 20 cm^3^ of n-hexane and refluxed for one hour and twenty minutes. The supernatant was carefully transferred into glass vials from which 4 cm^3^ was weighed for HPLC analysis.

### 3.5. Determination of Leaf Mineral Concentrations

Nitric acid was used for digestion of cation minerals. A portion of 200 mg of leaf sample was weighed into dry digestion tubes. Five millilitres of nitric acid was added, swirled and allowed to stand overnight. Tubes were placed into a digestion block with the temperature gradually increasing from room temperature to 120 °C over about 2 h with periodical swirling of each tube. Thereafter, the temperature was increased to 180 °C until about 0.5 cm^3^ of liquid remained. The digestion tubes were removed from the block and cooled at room temperature. The digest was diluted with ultrapure water, homogenized with a vortex mixer and allowed to stand for a few hours prior to analysis. The mineral concentration was determined using an inductively coupled plasma optical emission spectrometer (ICP-OES) (Optima 8000, Perkin Elmer).

### 3.6. Statistical Analysis

Data obtained were analyzed using the Statistical Package for Social Sciences (SPSS) version 21.0 for Windows. Analysis of variance (ANOVA) was used to compare means within the treatments while student’s *t*-test was used to compare means between treatments. Values were considered significant at *p* ≤ 0.05. All the data are expressed as mean ± standard deviation (SD).

## 4. Conclusions

This study compared the nutritional differencesbetween *Telfairia occidentalis* grown on gas flared and non-gas flared soil remediated with palm bunch biochar. Application of biochar was found to increase leaf moisture, ash, crude fibre and fat content of *Telfairia occidentalis* leaves with a decrease in leaf carbohydrate content of plants grown on gas flared soil. Increases in leaf vitamin and mineral content was observed in plants cultivated on gas flared soil treated with 13.9 t ha^−1^ and 7.1 t ha^−1^ of biochar. Biochar application could increase the mineral composition of vegetables and hence their nutritional quality.

## Figures and Tables

**Table 1:** Chemical composition of palm bunch biochar.

Mineral	Composition
pH	10.93 ± 0.11
Nitrogen (%)	1.35 ± 0.40
Calcium (%)	3.02 ± 0.01
Magnesium (%)	0.80 ± 0.02
Potassium (%)	1.01 ± 0.00
Phosphorus (%)	0.12 ± 0.00
Sodium (mg kg^−1^)	4.05 ± 0.11

Values are means ± standard deviation of duplicate determinations.

**Table 2:** Chemical composition of the soils used.

Parameters	Umudike (Control)	Egbema
pH	5.40 ± 0.71	5.45 ± 0.07
P (mg kg^−1^)	30.71 ± 0.84	20.82 ± 0.12
N (%)	0.13 ± 0.02	0.10 ± 0.01
OC (%)	1.48 ± 0.02	1.38 ± 0.08
OM (%)	2.55 ± 0.07	2.38 ± 0.14
Ca (cmol kg^−1^)	3.09 ± 0.16	2.15 ± 0.35
Mg (cmol kg^−1^)	0.77 ± 0.04	1.58 ± 0.03
K (cmol kg^−1^)	0.12 ± 0.00	0.11 ± 0.00
Na (cmol kg^−1^)	0.18 ± 0.00	0.23 ± 0.00
EA (cmol kg^−1^)	1.25 ± 0.07	0.87 ± 0.02
ECEC (cmol kg^−1^)	5.49 ± 0.01	5.20 ± 0.00
BS (%)	78.17 ± 0.03	83.05 ± 0.07 *

Values are means ± standard deviation of duplicate determinations. Value marked asterick (*) is significantlydifferent from control. P = available phosphorus, N = nitrogen, OC = Organic carbon, OM = Organic matter, Ca = calcium, Mg = magnesium, K = potassium, Na = sodium, EA = exchangeable acidity, ECEC = Effective cation exchange capacity, BS = base saturation.

**Table 3:** Effects of different application rates of palm bunch biochar applied to sandy loam soil on the vitamin composition of *T. occidentalis* leaves cultivated on gas flared and non-gas flared soils.

Soil	Biochar Treatment (t ha^−1^)				
	0	7.1	13.9	20.9	28
	**Vitamin A (mg g^−1^)**				
TONGFS	5.3 ± 0.5	20.7 ± 0.8 ^a^	30.2 ± 0.5	20.0 ± 0.1	10.2 ± 0.7
TOGFS	14.4 ± 0.9	24.9 ± 1.1 ^a^	15.5 ± 1.3 ^a^	16.6 ± 1.6 ^a^	14.7 ± 1.7 ^a^
	**Vitamin B_1_ (mg g^−1^)**				
TONGFS	0.9 ± 0.1	3.50 ± 0.1	5.00 ± 0.1	3.30 ± 0.2	1.70 ± 0.1
TOGFS	2.40 ± 0.1	4.10 ± 0.2 ^a^	2.60 ± 0.2 ^a^	2.80 ± 0.3 ^a^ *	2.50 ± 0.3 ^a^
	**Vitamin B_2_ (mg g^−1^)**				
TONGFS	31.6 ± 2.7	124.3 ± 4.9	181.2 ± 3.1	120.0 ± 0.1	61.1 ± 4.0
TOGFS	86.1 ± 5.4	149.2 ± 6.7 ^a^	93.0 ± 7.5 ^a^	99.3 ± 9.7 ^a^	88.4 ± 10.2 ^a^
	**Vitamin B_6_ (mg g^−1^)**				
TONGFS	4.0 ± 0.3	15.5 ± 0.6	22.6 ± 0.4	15.0 ± 0.1	7.6 ± 0.5
TOGFS	10.8 ± 0.7	18.7 ± 0.8 ^a^	11.6 ± 0.9 ^a^	12.4 ± 1.2 ^a^ *	11.1 ± 1.3 ^a^
	**Vitamin C (mg g^−1^)**				
TONGFS	1.1 ± 0.1	4.4 ± 0.2	6.5 ± 0.1	4.3 ± 0.2	2.2 ± 0.1
TOGFS	3.1 ± 0.2	5.3 ± 0.2 ^a^	3.3 ± 0.3 ^a^	3.5 ± 0.3 ^a^ *	3.2 ± 0.4 ^a^
	**Vitamin E (mg g^−1^)**				
TONGFS	6.3 ± 0.5	24.9 ± 1.0	36.2 ± 0.6	24.0 ± 0.4	12.2 ± 0.8
TOGFS	17.2 ± 1.1	29.8 ± 1.3 ^a^	18.6 ± 1.5 ^a^	19.9 ± 1.9 ^a^	17.7 ± 2.0 ^a^

Values are mean ± standard deviation of duplicate determinations. Values denoted with similar superscripts on the same roll and columns are statistically significant (*p* < 0.05). TONGFS = *T. occidentalis* grown on non-gas flared soil (Umudike), TOGFS = *T. occidentalis* grown on gas flared soil (Egbema).

**Table 4:** Effect of biochar remediation on the proximate composition of *T. occidentalis* leaves cultivated on gas flared and non-gas flared soils.

Soil	Biochar Treatment (t ha^−1^)				
	0	7.1	13.9	20.9	28
	**Protein (%)**				
TONGFS	11.78 ± 0.29	19.14 ± 0.55 ^a^	12.59 ± 0.30 ^a^	14.38 ± 0.18 ^a^	19.01 ± 0.03 ^a^
TOGFS	11.78 ± 0.29	18.81 ± 0.50 ^a^	12.67 ± 0.38 *	12.59 ± 0.30 ^a^	13.47 ± 1.80 *
	**Moisture (%)**				
TONGFS	4.19 ± 0.65	7.35 ± 0.29 ^a^	10.93 ± 0.76 ^a^	9.33 ± 0.22 ^a^	6.63 ± 0.41 ^a^
TOGFS	9.02 ± 0.01 *	11.24 ± 0.47 * ^a^	10.26 ± 0.71	8.83 ± 1.10 ^a^	11.03 ± 0.74 *
	**Ash (%)**				
TONGFS	9.77 ± 0.21	6.012 ± 0.18 ^a^	9.20 ± 0.33	7.29 ± 0.22 ^a^	9.1 ± 1.40
TOGFS	11.04 ± 1.16 * ^a^	9.24 ± 0.15 * ^a^	7.79 ± 0.49 * ^a^	6.07 ± 0.27 * ^a^	8.84 ± 0.44 ^a^
	**Crude fibre (%)**				
TONGFS	12.32 ± 0.18	9.28 ± 0.08 ^a^	12.39 ± 0.22 ^a^	13.89 ± 0.47	11.75 ± 0.17
TOGFS	9.95 ± 0.24 *	16.46 ± 0.94 * ^a^	15.71 ± 1.35 * ^a^	11.11 ± 0.62 *	12.96 ± 0.57 ^a^
	**Fat (%)**				
TONGFS	4.41 ± 0.36	7.47 ± 0.77 ^a^	3.96 ± 0.43	2.40 ± 0.08 ^a^	2.10 ± 0.08 ^a^
TOGFS	5.79 ± 0.47 *	3.39 ± 0.16 *	5.08 ± 0.19 *	5.85 ± 0.18 *	4.83 ± 0.08 *
	**Carbohydrate (%)**				
TONGFS	56.71 ± 0.50	50.74 ± 0.26 ^a^	50.94 ± 1.43 ^a^	52.72 ± 0.22 ^a^	51.40 ± 0.90 ^a^
TOGFS	52.42 ± 0.63 *	40.85 ± 0.91 * ^a^	48.48 ± 0.04 * ^a^	53.80 ± 1.57	48.87 ± 3.47 ^a^

Values are mean ± standard deviation of duplicate determinations. Values denoted with similar superscripts on the same roll and column are statistically significant (*p* < 0.05). TONGFS = *T. occidentalis* grown on non-gas flared soil (Umudike), TOGFS = *T. occidentalis* grown on gas flared soil (Egbema).

**Table 5:** Effect of different application rates of palm bunch biochar applied to sandy loam soil on the mineral composition of *T. occidentalis leaves* cultivated on gas flared and non-gas flared soils.

Soil	Biochar Treatment (t ha^−1^)				
	0	7.1	13.9	20.9	28
	**Calcium (mg g^−1^)**				
TONGFS	30.5 ± 1.2	12.3 ± 0.3	6.1 ± 0.4	26.2 ± 5.1	26.2 ± 5.1
TOGFS	52.4 ± 8.7	97.1 ± 0.9 * ^a^	74.3 ± 2.7 * ^a^	10.5 ± 0.2 ^a^	11.4 ± 0.3 ^a^
	**Magnesium (mg g^−1^)**				
TONGFS	8.4 ± 2.7	5.5 ± 0.0	3.0 ± 0.7	9.1 ± 1.4	9.1 ± 1.4
TOGFS	14.8 ± 4.8	4.0 ± 0.0 ^a^	30.7 ± 0.5 ^a^	5.0 ± 0.1 ^a^	4.6 ± 0.1 ^a^
	**Potassium (mg g^−1^)**				
TONGFS	2.7 ± 0.0	2.7 ± 0.2	1.6 ± 0.4	2.5 ± 0.7	2.5 ± 0.7
TOGFS	3.2 ± 0.4	4.0 ± 0.0 ^a^	4.0 ± 0.0 ^a^	2.0 ± 0.2 ^a^	1.9 ± 0.2 ^a^
	**Phosphorus (mg g^−1^)**				
TONGFS	1.8 ± 0.0	1.6 ± 0.0	1.6 ± 0.0	1.7 ± 0.2	1.7 ± 0.2
TOGFS	1.8 ± 0.4	2.0 ± 0.1 ^a^	2.0 ± 0.0 ^a^	1.6 ± 0.0 ^a^	1.6 ± 0.0 ^a^
	**Sodium (mg kg^−1^)**				
TONGFS	2.07 ± 0.11	3.79 ± 0.56	1.47 ± 0.06	1.93 ± 0.17	1.93 ± 0.17
TOGFS	1.70 ± 0.16	1.61 ± 0.04 ^a^	1.47 ± 0.06 ^a^	2.06 ± 0.19	1.54 ± 0.16

Values are mean±standard deviation of duplicate determinations. Values marked asterick (*) on the same column are statistically significant while those marked (^a^) on the same roll are significant (*p* < 0.05). TONGFS = *T. occidentalis* leaves grown on non-gas flared soil (Umudike), TOGFS = *T. occidentalis* leaves grown on gas flared soil (Egbema).
